# Dynamic Palpation for Diagnosing Clinically Covert Scaphoid Fractures

**DOI:** 10.7759/cureus.74537

**Published:** 2024-11-26

**Authors:** Nirmal K Sinha, Amit Bhardwaj, Ashutosh S Rao

**Affiliations:** 1 Orthopaedics, Manipal University College Malaysia, Melaka, MYS; 2 Orthopaedics, Sengkang General Hospital, Sengkang, SGP

**Keywords:** anatomy based palpation, covert scaphoid fracture, diagnosis of scaphoid fracture, dynamic palpation, pain provocation manoeuvres

## Abstract

The scaphoid is the most fractured carpal bone. In the initial workup, various clinical examinations are performed. However, the diagnosis can be confounding in the setting of clinically covert fracture cases. Routine physical examination findings may be equivocal in cases of proximal or distal pole fractures. Moreover, many commonly performed clinical tests are high in sensitivity but lag in specificity. Incorrect diagnosis can lead to overtreatment or undertreatment of this injury. To enhance the reliability of physical examination, dynamic palpation may be combined with static palpation. This report presents dynamic palpation at various wrist positions, based on cadaver anatomy studies of scaphoid and pain provocation manoeuvres of the wrist and hand as observed in various clinical studies. Tenderness thus elicited through these methods may help to confirm the diagnosis in the setting of clinically covert scaphoid fracture, where routine palpation findings are equivocal.

## Introduction

Background

The scaphoid is the most fractured carpal bone. Its incidence can be 10% of all hand fractures [1]. In the initial workup, various clinical examinations are performed, and many X-ray views are taken to confirm the diagnosis of fracture. Most performed clinical tests are high on sensitivity and low on specificity. The incidence of polar fractures can be as much as 48% of total scaphoid fractures [2]. Because of their anatomical location, they are difficult to access and properly palpate. Hence, findings of physical examination are likely to be equivocal in many polar fracture cases. Moreover, the diagnosis can be confounding in clinically covert or radiologically occult fracture cases. Some patients may not have pain over the scaphoid, even when a well-defined fracture is seen on the radiograph [3]. Conversely, X-rays may not show a distinct fracture line in some cases of scaphoid fracture [4-6]. In his systematic review studies of 42 studies, Bäcker et al. observed that up to 16% of fracture cases were missed, where X-rays were inconclusive [4]. Incorrect diagnosis can result in overtreatment or undertreatment of this injury. Overtreatment may affect the quality of living and lead to loss of working hours. Missing or late diagnosis of this injury is associated with serious complications like avascular necrosis, non-union, wrist osteoarthritis, and scaphoid non-union with advanced carpal collapse. These considerations highlight the need for a more reliable approach to physical examination in clinically covert scaphoid fracture cases. Two cadaver-based studies on scaphoids have greatly contributed to our understanding of the surface anatomy of scaphoids [7,8]. The enhanced palpability of the scaphoid in various wrist positions, as shown through these studies, may be critical in improving the reliability of the physical examination of the scaphoid.

Objective

The objective of this paper is to revisit and formalise steps of dynamic palpation of the scaphoid, based on cadaver-based anatomical studies and physical manoeuvres of the wrist and thumb, with the purpose of enhancing the sensitivity and specificity of clinical examination of the scaphoid.

## Technical report

Suggested method of dynamic palpation

The authors suggest steps of dynamic palpation of the scaphoid in various wrist positions, so that palpability of the bony surface of the scaphoid is maximized. Furthermore, tenderness provoked by wrist and thumb movement may enhance the reliability of bony palpation.

**Figure 1:**
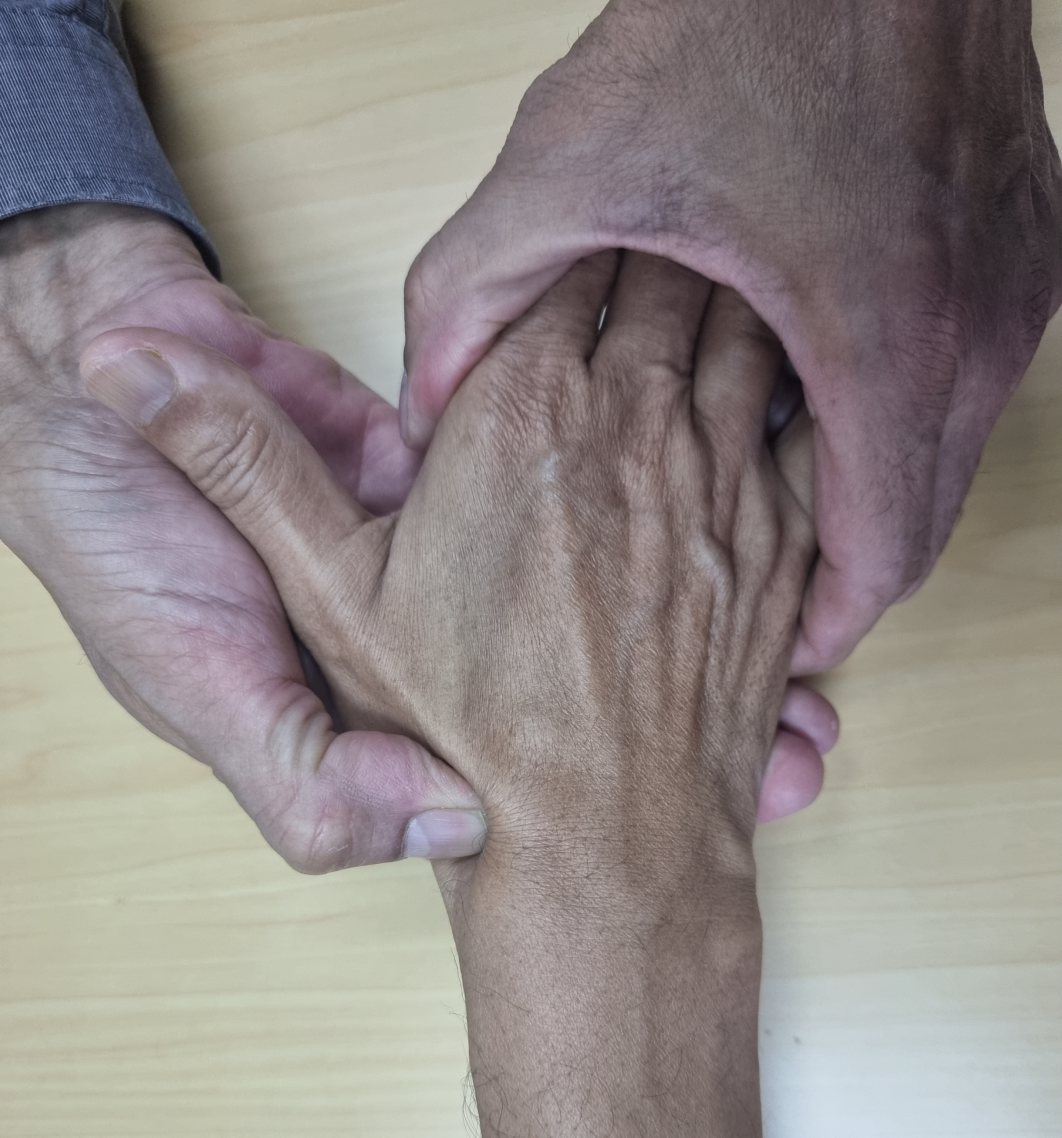
Palpation of anatomical snuff box with wrist in neutral position

**Figure 2:**
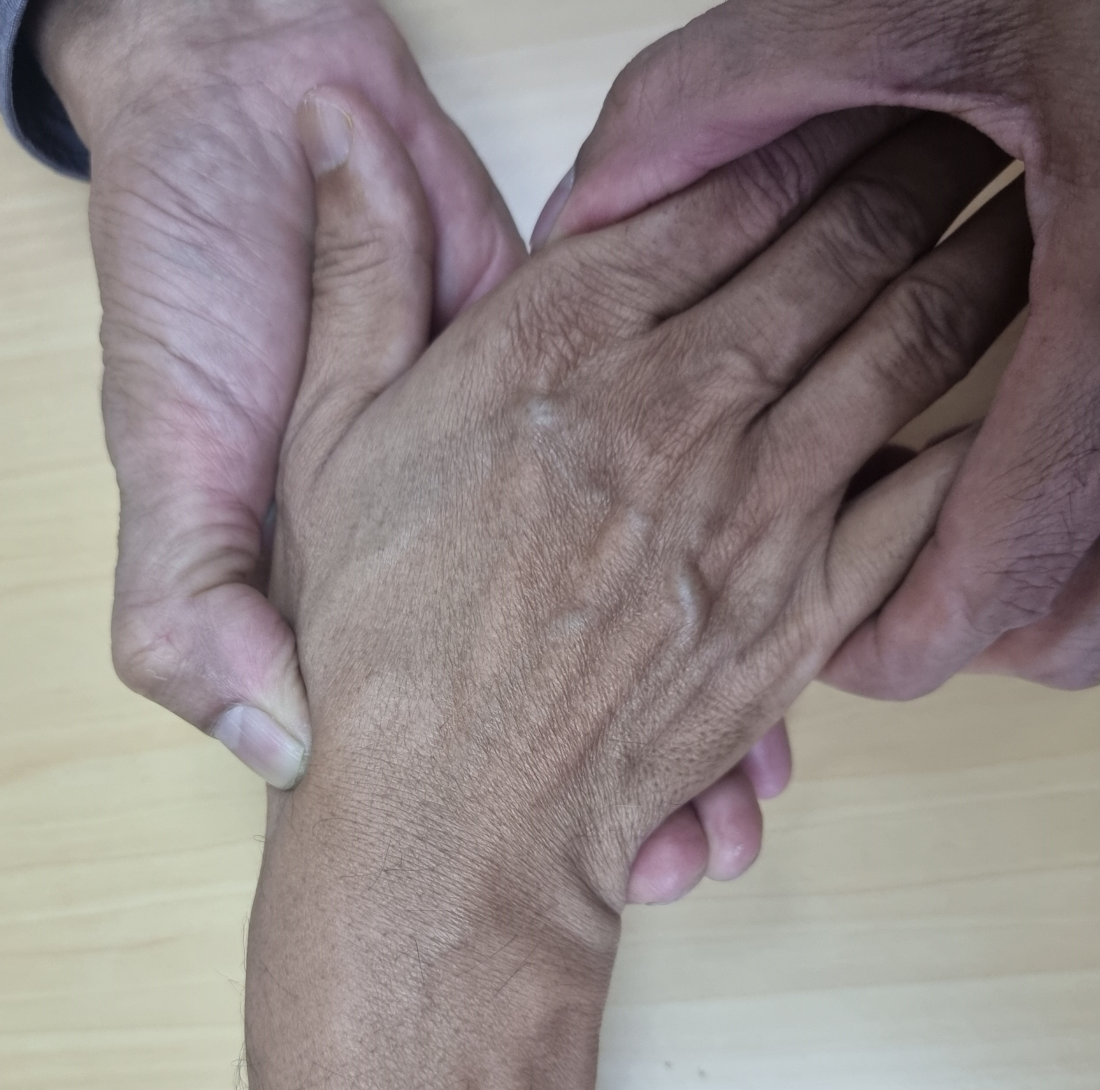
Dynamic palpation while moving wrist to full ulnar deviation

**Figure 3:**
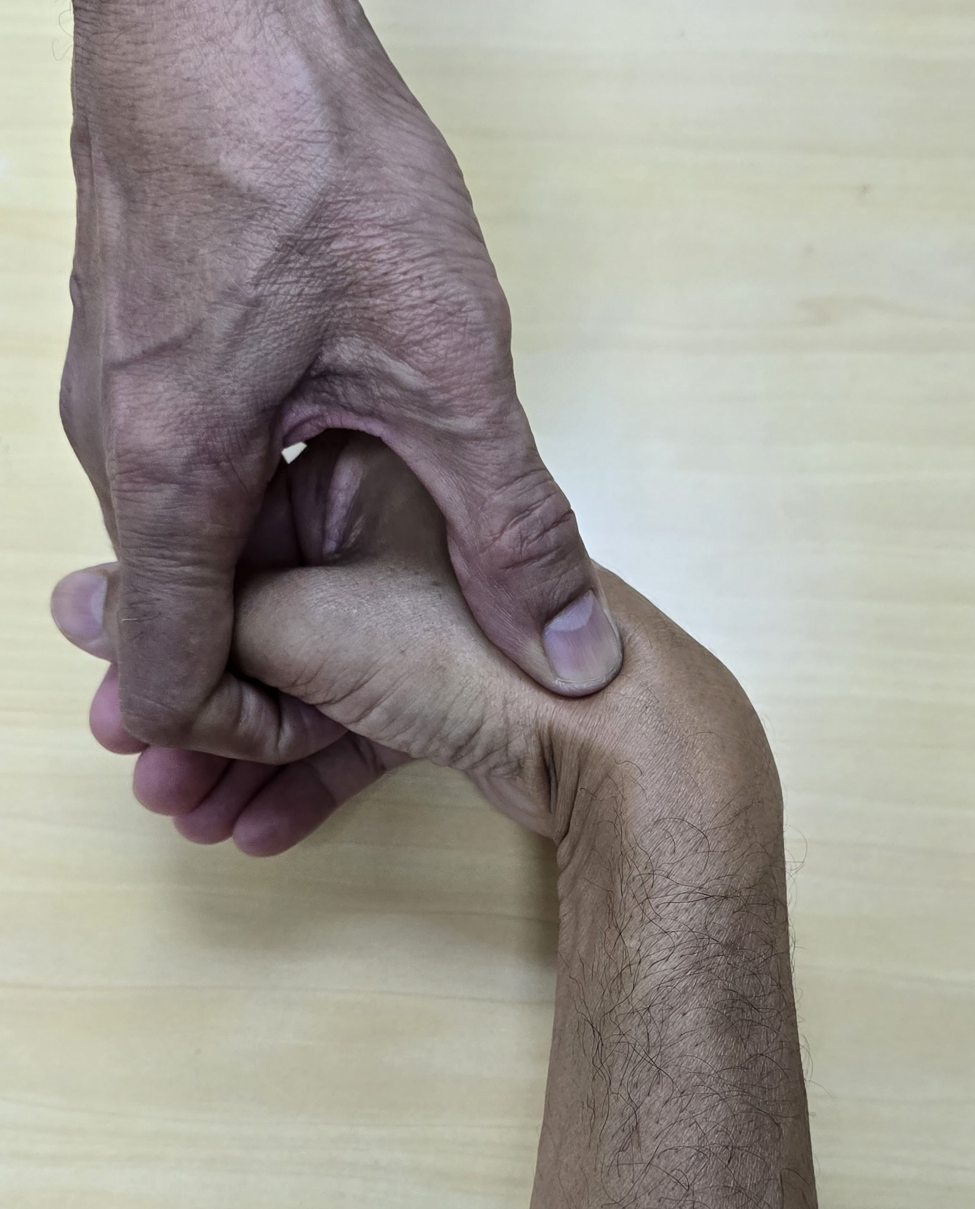
Flex the wrist while palpating for the proximal pole of scaphoid

The steps to perform dynamic palpation are mentioned below:

1. Start bony palpation of the anatomical snuff box with the wrist in a neutral position (Figure 1).

2. Continue the palpation of the scaphoid while moving the wrist to maximum ulnar deviation. The thumb can also be flexed along with wrist movement (Figure 2).

3. Bring the wrist back to the initial position of neutral extension (Figure 1).

4. Flex the wrist maximally from the neutral position while palpating the most proximal pole of the scaphoid (Figure 3).

5. Perform scaphoid tubercle tenderness test or Watson’s scaphoid shift test to demonstrate tenderness of distal pole fracture.

6. Perform other pain provocation tests like thumb compression tests to further reinforce the diagnosis.

## Discussion

The most informative step in the physical examination is to elicit a point bony tenderness at the site of fracture [7,9]. Palpation performed to elicit tenderness can be static (where tenderness is produced by palpating the involved bone) or dynamic (where the involved bone is stressed, or the wrist is moved to produce a painful response). The following methods of palpation are static in nature, and they are commonly used in the initial workup: (1) Anatomical snuffbox tenderness; (2) volar palpation of scaphoid tubercle; (3) bi-digital palpation of scaphoid.

In cases with equivocal findings, dynamic palpations are more likely to elicit tenderness. These tests inflict compression, rotatory or angular force on the body of the scaphoid [7]. Compression or torque produced by these forces may produce stress or micromotion at the site of cortical discontinuity and thus unmask tenderness. The common ones under this category, as mentioned in various studies, are as follows: (1) Thumb axial compression [10]; (2) pain over the scaphoid on the ulnar deviation of the wrist [9,11]; (3) pain provocation on thumb movement [5]; (4) Watson’s scaphoid shift test [7]; (5) ulnar deviation of the pronated wrist [9].

Anatomical snuffbox tenderness is considered as the single most important test to detect a fracture. Mallee et al. did a meta-analysis of a clinical diagnostic evaluation of various clinical tests and observed that anatomical snuff box tenderness was the most sensitive clinical test [12]. Chen introduced the scaphoid compression test in a study on 52 patients and observed that it is a reliable test, and it can be performed while the patient’s wrist is in a cast [10]. In a prospective study on 221 patients, Grover found scaphoid axial compression tenderness to be the most accurate test with a sensitivity of 100% and a specificity of 80% [13]. In a study of 250 cases of suspected scaphoid fracture cases, Parvizi et al. observed that at the initial examination of anatomical snuffbox tenderness, thumb axial compression and volar palpation of scaphoid tubercle were 100% sensitive for detecting scaphoid fracture with specificities of 9%, 30% and 48% respectively [5]. The clinical signs used in combination, within the first 24 hours following injury, produced 100% sensitivity and an improvement in the specificity to 74%. Hence, the study concluded that no test was reliable if done alone and that they should be combined to enhance the reliability of physical examination.

As proposed in this article, dynamic palpation may provoke a more painful response when moving the wrist to a specific wrist position. The concept is supported by cadaver-based anatomical studies and pain provocation signs related to wrist movements.

Young and Giachino studied scaphoid anatomy in various wrist positions on three cadavers [7]. Later, Giugale et al. studied the surface anatomy of the scaphoid by digitising and developing three-dimensional images of the scaphoid in various wrist positions [8]. These cadaver-based studies show that the maximum palpable area of the scaphoid is obtained with the wrist in neutral extension and maximum ulnar deviation. In this position, the waist and proximal pole except for the most proximal part of the scaphoid are most palpable through the anatomical snuff box. Hence, this position may be optimal for detecting tenderness from the scaphoid waist and most proximal pole fractures. The studies further show that the most proximal part of the scaphoid is accessible with the wrist in a neutral position and with maximum flexion of the wrist. For a more reliable examination of the distal pole, scaphoid tubercle tenderness or Watson’s scaphoid shift test should be performed [7].

Various studies have demonstrated that bony tenderness of the scaphoid is elicited by movements of the wrist and thumb. This pain may be provoked by stressing the site of cortical discontinuity or micro-movement at the fracture site. In a study involving 73 patients, Powell et al. observed that pain elicited by mere movement of the wrist into ulnar deviation was 83% to 100% sensitive for detecting scaphoid fractures [9]. The test had a 52% positive predictive value of 52% and a negative predictive value of 100%. Parvizi et al. also found that thumb movement had a sensitivity of 69% and a specificity of 66% for detecting scaphoid fractures [5].

## Conclusions

Dynamic palpation based on the surface anatomy of the scaphoid at various wrist positions and pain provocation manoeuvres of the wrist and hand may elicit tenderness at the site of cortical discontinuity in the setting of clinically covert scaphoid fracture. However, palpation of the scaphoid in various wrist positions, as proposed in this article, is based on cadaveric anatomy studies and requires validation through clinical studies involving scaphoid fracture cases.
